# Beyond Profit: The Ethical Compass of Banting and Salk in Medical Innovation

**DOI:** 10.7759/cureus.57698

**Published:** 2024-04-06

**Authors:** Sohilkhan R Pathan, Kruti B Sharma

**Affiliations:** 1 Clinical Research Services (CRS), Bhanubhai and Madhuben Patel Cardiac Centre, Shree Krishna Hospital and Medical Research Centre, Bhaikaka University, Anand, IND

**Keywords:** altruism, polio vaccine, insulin, jonas salk, frederick banting

## Abstract

In this editorial, we explore the profound contributions of scientists Frederick Banting and Jonas Salk to medical science. Their discoveries of insulin and the polio vaccine, respectively, revolutionized healthcare and exemplified a moral commitment to prioritize human welfare over financial gain. Banting and Salk's decision not to patent their life-saving inventions underscored a noble ethos in pharmaceutical innovation, emphasizing a dedication to the greater good. Their legacies challenge contemporary pharmaceutical practices, urging a reevaluation of values to prioritize compassion and societal impact. This abstract highlights the enduring significance of Banting and Salk's legacies and their profound impact on medical science and society.

## Editorial

In the annals of medical history, there stand towering figures whose contributions transcend the boundaries of scientific achievement. Among them, Frederick Banting and Jonas Salk shine as beacons of altruism, their legacies woven with selflessness and an unwavering dedication to humanity. Their discoveries, insulin and the polio vaccine, respectively, stand not only as monumental milestones in medical science but also as a testament to a moral imperative often overlooked in today's profit-driven world. Banting's breakthrough in the discovery of insulin in 1921 revolutionized the treatment of diabetes, rescuing countless lives from the clutches of this debilitating disease [1]. Similarly, Salk's development of the polio vaccine in the 1950s heralded a new era in the fight against one of the most feared diseases of the time, offering hope to millions worldwide [2].

What sets these scientific pioneers apart is not merely their scientific acumen but their profound understanding of the ethical responsibility that accompanies groundbreaking discoveries. Unlike many in their position, Banting and Salk chose not to patent their life-saving inventions, recognizing that the value of their work lay not in financial gain but in the alleviation of human suffering. Their actions epitomize the noble ethos that should underscore pharmaceutical innovation - a commitment to serve the greater good. In a world where profit often takes precedence over compassion, their example serves as a poignant reminder of the true purpose of scientific endeavor: to improve the lives of others. It is crucial to acknowledge that Banting and Salk's decisions were not made in isolation. They were shaped by a deep-seated sense of empathy and a profound understanding of the privilege and responsibility that come with scientific discovery. Their humility in recognizing that their work belonged to humanity, not for their personal gain, is a lesson that resonates profoundly in today's world.

In an age where the pharmaceutical industry is frequently criticized for prioritizing profit margins over public health, the legacies of Banting and Salk serve as a moral compass. Their example challenges us to reconsider the values that underpin medical research and innovation. It reminds us that true progress is measured not only in scientific breakthroughs but in the tangible impact they have on the lives of those in need. As we celebrate the achievements of Banting and Salk, let us also reflect on the values they embodied - the values of compassion, integrity, and a steadfast commitment to the common good. Let us honor their legacy by striving to emulate their selflessness and by championing a culture of scientific innovation that prioritizes human welfare above all else.

In doing so, we can ensure that the spirit of Banting and Salk lives on, inspiring future generations of scientists to harness the power of discovery for the betterment of all humankind. For, in the end, it is not the accolades or the financial rewards that define a scientist's legacy, but the lives they touch and the suffering they alleviate through their tireless dedication to the greater good.
